# Wear Properties of Nitride-Bonded Silicon Carbide under the Action of an Abrasive Soil Mass

**DOI:** 10.3390/ma14082043

**Published:** 2021-04-19

**Authors:** Jerzy Napiórkowski, Klaudia Olejniczak, Łukasz Konat

**Affiliations:** 1 Department of Construction, Vehicle and Machine Operation, The Faculty of Technical Sciences, University of Warmia and Mazury in Olsztyn, M. Oczapowskiego 11, 10-719 Olsztyn, Poland; napj@uwm.edu.pl; 2 Department of Vehicle Engineering, Faculty of Mechanical Engineering, Wrocław University of Science and Technology, Wybrzeże Wyspianskiego 27, 50-370 Wrocław, Poland; lukasz.konat@pwr.edu.pl

**Keywords:** nitride-bonded silicon carbide, abrasive wear-resistant steel, padding weld, abrasive soil mass, grain size distribution

## Abstract

Nitride-bonded silicon carbide is an alternative to steels resistant to abrasive wear. This paper presents the results of a nitride-bonded silicon carbide (SiC) wear test in diverse soil conditions. The test was performed on a “spinning bowl” test stand on three soil types: loamy sand, light loam and ordinary loam. The results were referred to the wear test for materials used to make parts working soil mass, i.e., abrasive wear-resistant steel, boron steel and C + Cr + Nb padding weld. The abrasive wear resistance of silicon carbide was shown to depend on the grain size distribution of the soil being worked. Silicon carbide showed the highest resistance in light soil. However, the padding weld showed higher wear resistance in the other soil conditions. Nitride-bonded silicon carbide had higher wear resistance than the steels under study in all of the soils. These findings are supplemented by an analysis of the condition of the worked surfaces after friction tests. The dominant wear methods in all abrasive masses were micro-cutting and furrowing.

## 1. Introduction

Because of its physicochemical properties, silicon carbide (SiC) is increasingly often used in structural elements, not only in construction but also in machine building. Its main characteristics include:

- Low density (approx. 3.1 g/cm^3^) [1,2];
- High hardness (approx. 2500 HV);
- High abrasion resistance;
- Chemical resistance, also at high temperatures;
- Thermal decomposition at temp. above 2000 °C;
- High thermal conductivity;
- Resistance to thermal shocks (Δ ~ 250 °C);
- Low thermal expansion (4.3–5.8 × 10^−6^ K).

Due to the low coefficient of friction and resistance to abrasive wear, ceramic materials are used, among others, for containers for storing bulk materials, including abrasive materials, pipes transporting materials even at high temperatures, crucibles for melting aluminium, for lining resistance furnaces and kilns and furnaces for glass melting and casting. It is also used in heavy-duty applications such as car brakes, car clutches and, due to its temperature properties, for coating friction surfaces operating at high temperatures; for example, the side surfaces of engine cylinders and as thermal shields in spacecraft.

In many of these applications, silicon carbide is abraded by natural materials containing abrasive particles, including soil. Characteristic features of silicon carbide also include mechanical strength. They best transmit compressive loads, but they have a low tolerance for stretching and bending. Development of innovative materials, such as composites with graphene (PNB) [3] or zirconium ceramics (ZrO_2_) [4], opens a considerable perspective for silicon carbide.

The resistance to brittle cracking is a limitation of their resistance to tribological wear. Grain size distribution is also of great importance because resistance to tribological wear decreases with increasing grain size of the abraded material. The presence of reinforcing phase grains is a decisive factor in abrasion resistance [5]. Starting with the need to improve the properties of ceramic material from silicon carbide, a technology of nitride-bonded silicon carbide (SiC + Si_3_N_4_) production was developed. Compared to other technologies of sintered silicon carbide production, materials from nitride-bonded silicon carbide are characterised by high abrasion resistance and also by increased brittle cracking resistance, as well as by increased impact wear and friction wear resistance.

The literature usually identifies three types of tribological wear [6]:

- Adhesive—occurs at a relatively low relative velocity of interacting surfaces and at high unit pressures in the areas of the actual contact surfaces;
- Erosive—results from small abrasive particles hitting against the material surface; the particles can be transported both by gases and by liquids;
- Abrasive wear—is associated with high roughness of the rubbing surface or the presence of loose abrasive material between the surfaces, which results in the loss of material caused by micro-cutting, scratching or grooving [7].

The complexity of the processes leading to tribological wear and tear makes experimental methods of abrasion resistance testing very important in determining this characteristic. The selection of the right measurement method is based on an analysis of the item operating conditions. The assessment of wear resistance is usually based on the use of friction, grinding or impact systems. The known methods of determining abrasion resistance include the contact and surface abrasion methods used to assess adhesive and abrasive wear and the impact wear methods giving values of erosive wear [8,9].

Abrasive wear of brittle materials is often described with an equation which is used to calculate the volume of cut-out material along a scratch formed during a time unit [10] or a loss of mass after friction tests [11]. This issue has been tested in dry conditions for many years [8,9,12,13,14,15,16,17].

Due to its versatility, steel is the basic construction material used for working pieces. However, taking into account, on the one hand, the manufacturing cost and, on the other hand, the declared abrasion resistance, nitride-bonded SiC materials can become a substitute for these materials. To date, no information has been found in the literature on the wear pattern of silicon carbide-based materials compared to materials commonly considered as resistant to wear in natural abrasives. The available literature reports show the topicality of the issue of new silicon carbide materials wear [3,4,15,18,19,20,21,22,23,24].

The objective of this study is to assess the wear properties of nitride-bonded silicon carbide compared to steel and padding weld commonly used in a metal–mineral tribological pair.

## 2. Methods and Materials

### 2.1. Wear Resistance Test

The chemical composition was analysed by the spectral method with a GDS500A glow discharge emission analyser (LECO Corporation, St. Joseph, MI, USA) manufactured with the following parameters: U = 1250 V, I = 45 mA, argon. The results were an arithmetic average of five measurements. The hardness of the samples was tested by the Brinell method as per the standard PN-EN ISO 6506-1:2008P with a hardness tester (Zwick Roell Gruppe, Ulm, Germany) with a 2.5 mm sintered carbide, at the load of 1875 kgf acting for 15 s. The hardness of the padded layer was measured by the Vickers method as per standard PN-EN ISO 6507-1:1999. The measurements were performed with a hardness tester, at the load of 1 kg (9.807 N) for 15 s.

The following testing equipment was used in the experiment:

- For macroscopic tests and for surface assessment after the friction tests—digital microscope KEYENCE VHX-6000 (Keyence Corporation, Mechelen, Belgium)
- For microscopic tests under an optical microscope—optical microscope (Nikon Eclipse MA200, Nikon Corporation, Tokyo, Japan). The observations were conducted at the magnification of 100 to 5000×;
- The microstructure images were taken with a digital camera (Nikon DS-Fi2, Nikon Corporation, Tokyo, Japan) with the use of NIS Elements software (Nikon Corporation, Tokyo, Japan);
- Examinations by scanning electron microscopy and chemical composition microanalyses were performed with a scanning electron microscope (SEM) JSM-5800LV (JEOL Ltd., Tokyo, Japan) coupled with an X-ray analyser (Oxford Instruments plc, Abingdon, UK). The accelerating voltage of 20 and 25 kV was used in the experiment. Microstructure observations were conducted in material contrast with SE and BSE detectors. Specimens were sprinkled with amorphous carbon before the microscopic observations;
- For examination of surface roughness after friction tests—a laser scanning confocal microscope 3D.

**Figure 1:**
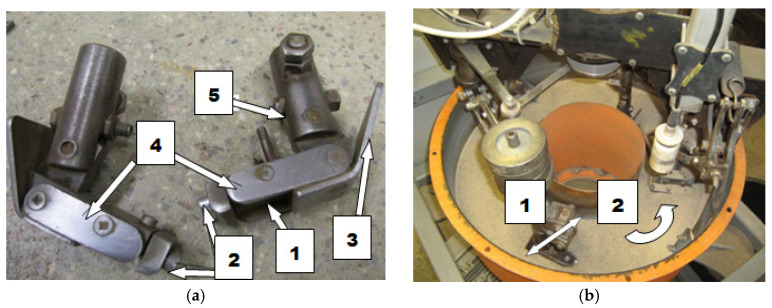
The “spinning bowl” type laboratory wear testing stand: (**a**) feet mounted on the stand arms with a designated place for sample fixing; (1) place of sample fixing, (2) screws holding the sample, (3) front skid, (4) side sample cover, (5) screw for changing the rake angles; (**b**) fragment of the stand during the operation; arrow (1) shows the direction of the sample movement, arrow (2) shows the direction of the bowl rotation.

Abrasive wear resistance tests of the steel under analysis were performed by the spinning bowl method with a MZWM-1 device. Figure 1 shows a diagram of the general structure and principle of operation of the device. Each specimen covered the total distance of 20,000 m during the test at the speed of approx. 1.7 m/s and unit pressure of 67 kPa. The specimen weight was determined every 2000 m on a laboratory balance with an accuracy of 0.0001 g, after they were cleaned in an ultrasound washer. The soil mass was replaced with a new portion and the initial mass parameters were established at the time. The specimens moved with oscillatory movement along the friction path. The abrasive mass pH ranged from 6.6 to 6.9. The following natural soil types were used in the experiment: loamy sand, light loam and ordinary loam and referred to light, medium and heavy soil, respectively. The soil moisture content ranged from 10% for loamy sand to 15% for ordinary loam, which corresponds to humid soil. The soil moisture content was determined by measuring the weight of the solid phase dried at 105 °C. The grain size distribution was determined by laser diffraction with a laser diffraction particle size analyser (Mastersizer 2000, Malvern, UK) as per standard ISO 13320. Characteristics of the abrasive soil mass taken for the study are shown in Table 1.

Specimen mass wear and its intensity were determined from the following data: weight wear of a specimen;

(1) $$Z_{pw}=m_{w}-m_{i}\;\left[g\right]$$

where: *m_w_*—initial sample mass before friction (g), *m_i_*—specimen mass after friction distance of *S* (g), intensity of mass wear;

(2) $$I_{pw}=\frac{Z_{pw}}{S}\;\left[\frac{g}{\mathrm{km}}\right]$$

where: *S*—friction distance (km).

The wear resistance of the test materials was compared using the wear resistance index *K_b_* [10]. Nitride-bonded silicon carbide was used as the standard material.

(3) $$K_{b}=\frac{\frac{Z_{Vw}}{S_{Tw}}}{\frac{Z_{Vb}}{S_{Tb}}}=\frac{Z_{Ww}\times \rho_{b}\times S_{Tb}}{Z_{Wb}\times \rho_{w}\times S_{Tw}}$$

where: *Z_Vw_*—volume wear of the standard material, *Zv_b_*—volume wear of the tested material, *Z_Ww_*—mass wear of the standard material, *Z_Ww_*—mass wear of the tested material, *S_Tw_*—friction distance of the standard material, *S_Tb_*—friction distance of the tested material, *ρ_w_*—density of the standard material, *ρ_b_*—density of the tested material.

### 2.2. Materials

Specimens of all the materials under test were taken in the shape of 30 × 25 × 10 mm cuboids by methods that ensure their unchanged structure. The specimens were cut out with a high-power water jet with abrasive material.

**Figure 2:**
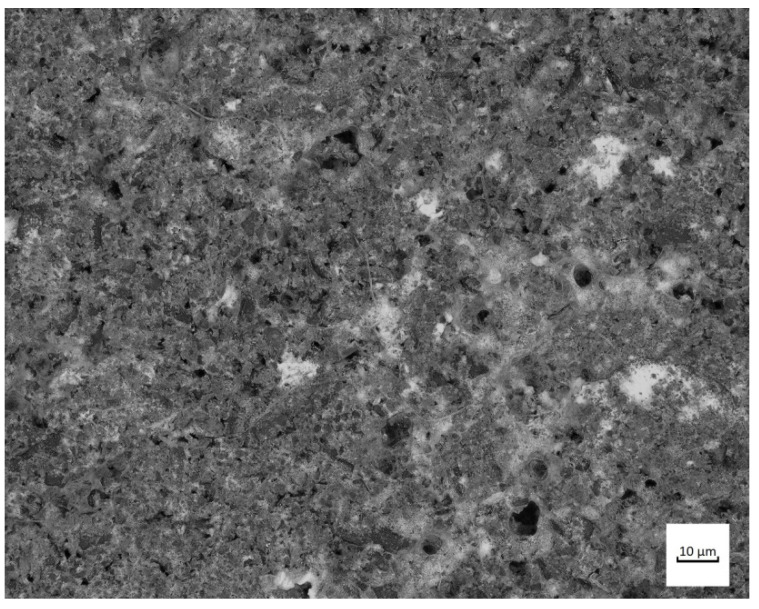
Surface of nitride-bonded silicon carbide.

This study analysed nitride-bonded silicon carbide SiC–A 80. Ten-millimetre-thick plates of nitride-bonded silicon carbide were made by reactive sintering at 1300 °C in a nitrogen atmosphere. Reactive sintering is a method which consists of producing a binding phase in the material being sintered as a result of a reaction between carbon (or its precursor) and solid or liquid silicon. Materials made in this manner often contain remains of unreacted substrates. Several different phases can be formed in the C-Si-O-N system: silicon carbide, silicon nitride, silicon oxynitride and silica. The SiC–A 80 surface is shown in Figure 2, and the chemical composition and the characteristics of the carbide under study are presented in Table 2 and Table 3. On the basis of the certificate, the conformity of the product declared by the manufacturer was found. The chemical analysis of the silicon carbide was carried out in accordance with the BS EN 12698-1:2007 Chemical analysis of nitride bonded silicon carbide refractories. Chemical methods standard.

**Figure 3:**
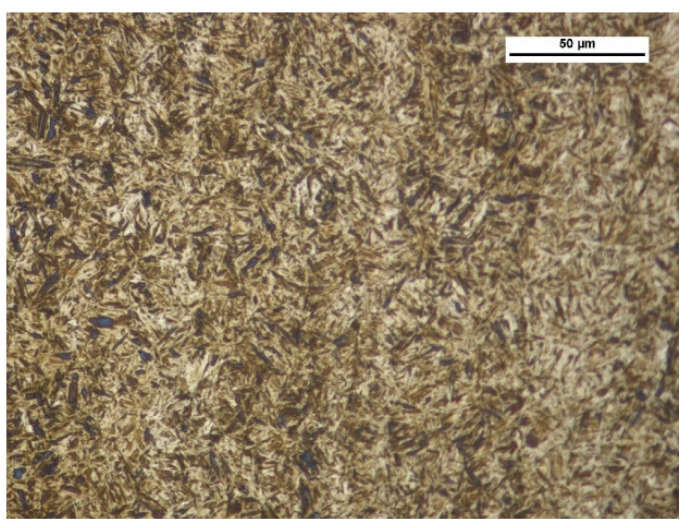
XAR 600 steel microstructure—tempered martensite. Magnitude ×500, etched with 3% HNO_3_ (Mi1Fe), scanning microscopy.

**Figure 4:**
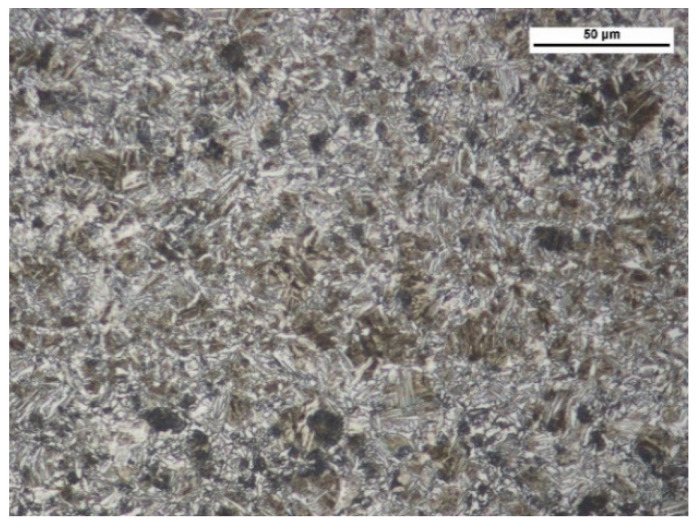
B27 steel microstructure—semi-martensitic with fine carbide grains. Magnitude ×1000, etched with 3% HNO_3_ (Mi1Fe), scanning microscopy.

The wear test involved two types of abrasive wear-resistant steel: XAR 600 (Figure 3) (samples were taken from 10 mm thick sheets, provided directly by the manufacturer) and boron steel B27 (Figure 4) (samples were taken from working parts of agricultural machines, made of 0.01 m steel sheets and subsequently subjected to thermal treatment) and F-61 padding weld. The materials were subjected to grinding treatment, after which the steel roughness was Ra = 0.25 µm and Ra = 0.34 µm for the padding weld. The XAR 600 steel microstructure comprises mainly fine lath-tempered martensite. The structure was also seen to contain numerous bands. Martensite laths were found to contain some separate carbide particles. The structure of B27 steel is typical of quenched and low-temperature tempered steel. It has a semi-martensitic structure with fine carbide grains within the martensite. These are MoC, Cr_7_C_3_, Cr_23_C_6_, Cr_3_C_2_ coherent carbides and M_23_B_6_ borides [6]. 

**Figure 5:**
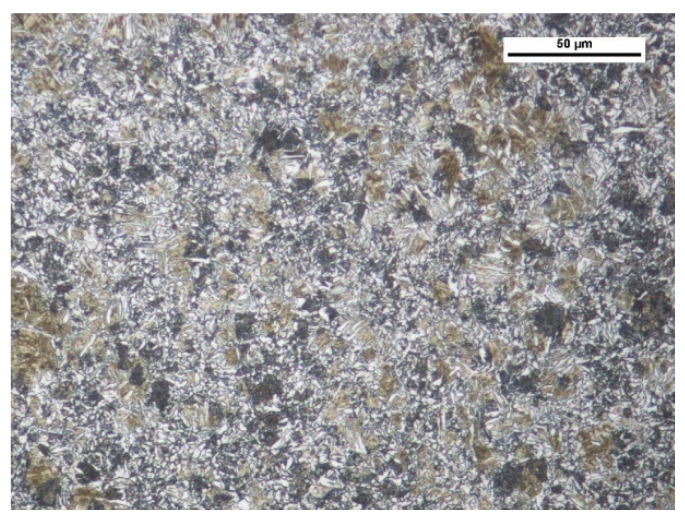
38GSA steel microstructure—fine dispersed perlite with grains of ferrite and martensite. Magnitude 500×, etched with 3% HNO_3_ (Mi1Fe).

A padded layer applied onto machine part surfaces, bound to the macro- and microstructure are one of the main trends in improving the abrasive wear resistance in a soil mass. An analysis of the equilibrium graphs and studies of the phase structure and padded weld microstructure was the starting point for the study. The padded layer was obtained by applying on 38 GSA low-alloy martensitic steel (Figure 5) a coated electrode made of Fe–Cr–C alloys with an addition of niobium (chemical composition of the first layer) (Table 4) with the welding current set at 100 A. The carbon content was 5.2%, chromium content—29%. Chemical composition of 38 GSA steel: C—0.38, Mn—1.12, Cr—0.15, Si—0.85, P—0.11, Ti—0.09, Al—0.04.

**Figure 6:**
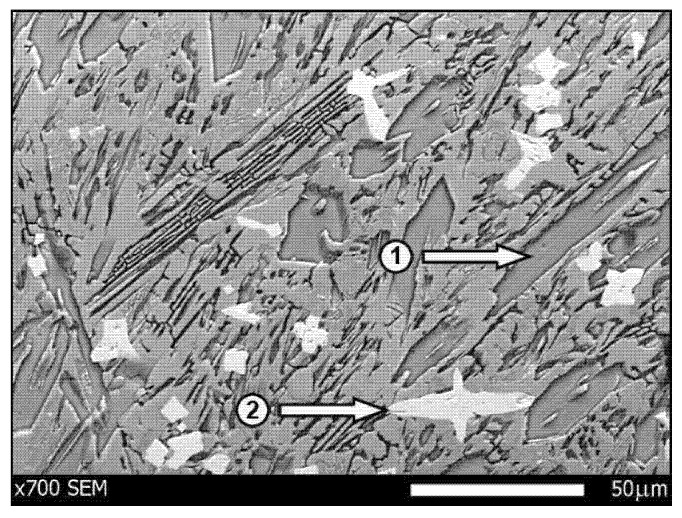
Microscopic image of the padded layer F-61. Microstructure of the padded layer with grains of chromium (1) and niobium (2) carbides. Etched with 3% HNO_3_ (Mi1Fe) and electrolytically with chromic acid, scanning microscopy.

Chromium and the other alloy additions stabilise alloy ferrite, while at the same time forming carbide phases of a complex structure (primary and secondary, e.g., Cr) or simple (e.g., Nb). Therefore, the phase structure of the padding weld under study, which solidifies in conditions close to equilibrium, consists of alloy ferrite and alloy Cr + Nb carbides (Figure 6).

## 3. Analysis of Results

**Figure 7:**
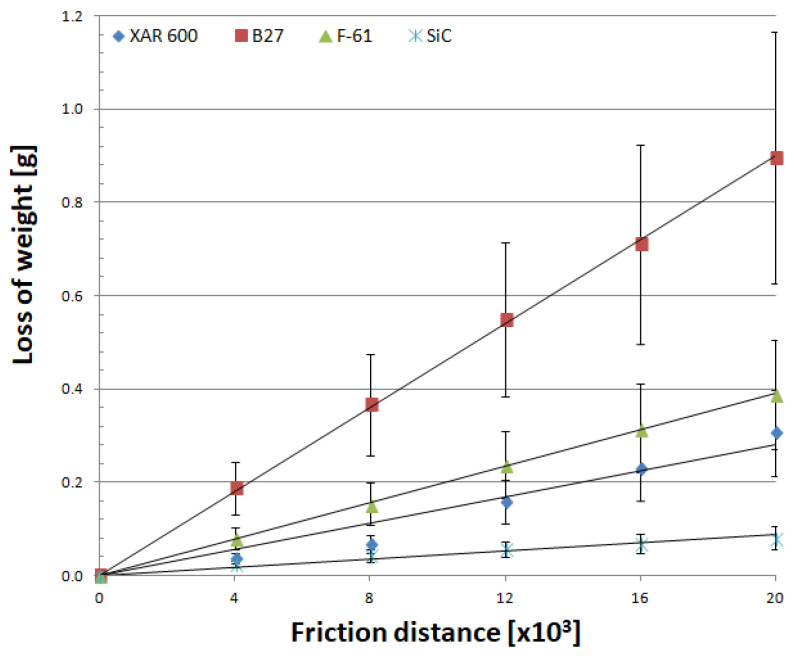
Mass wear as a function of friction distance for materials in light soil.

**Figure 8:**
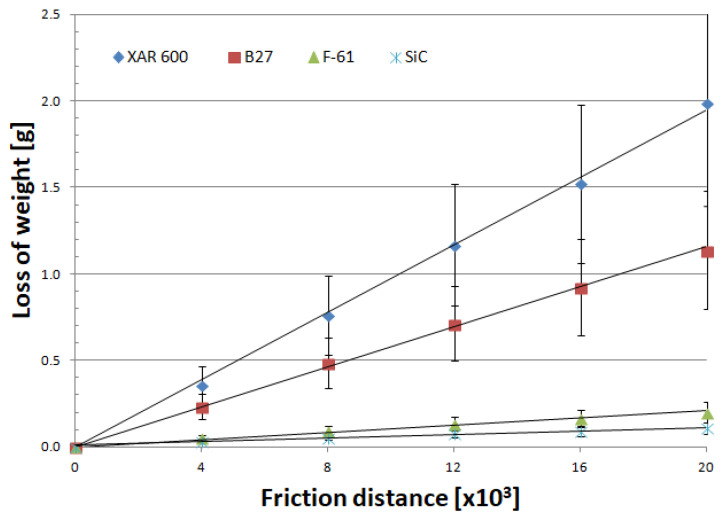
Mass wear as a function of friction distance for materials in medium soil.

**Figure 9:**
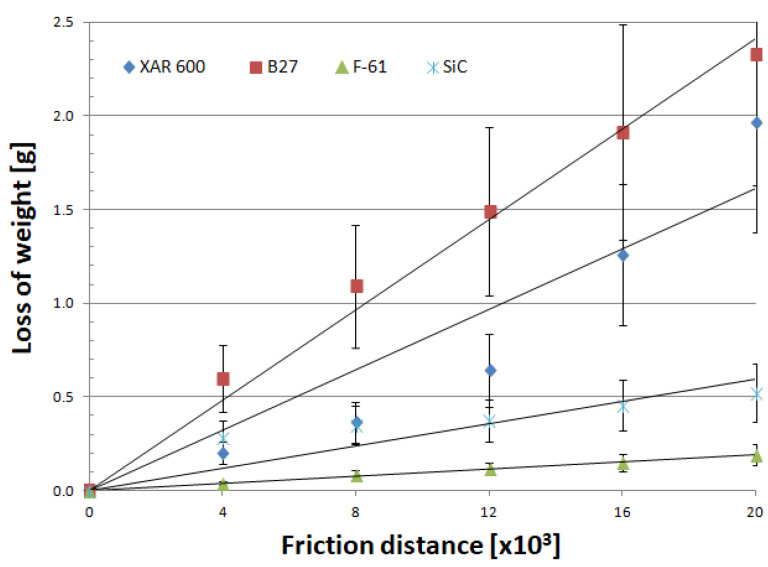
Mass wear as a function of friction distance for materials in heavy soil.

Mass wear as a function of friction distance for materials is well described by linear equations, regardless of the soil type (Figure 7, Figure 8 and Figure 9). As in other studies [19,25,26,27,28,29], soil grain size distribution has a fundamental effect on the mass wear of the materials under study (Table 5). The wear of nitride-bonded silicon carbide in abrasive soil mass also depends on the soil grain size distribution.

**Figure 10:**
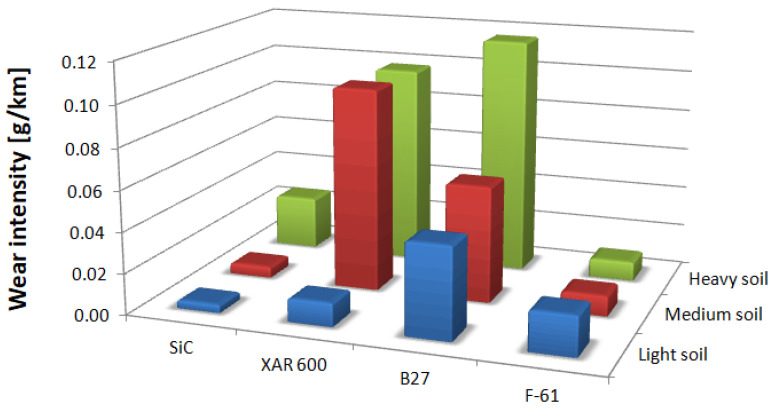
Unit wear of tested materials in individual soils.

Analysis of the wear characteristics of silicon carbide as a function of the soil type regarding the top layers commonly used on soil working parts was the main issue discussed in the paper. The results show that the steel type for working parts should be chosen with the type of soil mass to be worked in mind. Silicon carbide proved to be the most resistant to mass loss in light soils. The carbide wear resistance in medium soils was similar to that of the layer obtained by applying the F-61 padding weld. The lowest wear in heavy soil was determined for the F-61 padding weld. The wear of nitride-bonded silicon carbide was much lower than that of boron-containing steel in all the soils under study. This is well illustrated by the unit wear for nitride-bonded silicon carbide in different soil conditions, shown in Figure 10. The silicon carbide wear in heavy soil was nearly five times greater than in medium soil and more than six times greater than in light soil. The anti-wear properties of the other top layers were found to change depending on the soil type. The tests were performed in soils of the same moisture content, slightly acidic, at the same friction speed and under the same load. Soils can also be identified for each top layer under study in which the wear process will be the least intensive. Except for silicon carbide, the lowest wear in sandy (light) soils was observed for XAR 600 and B27 steels (Figure 10). It was observed that the wear of these top layers increases with increasing silt and dust content in the soil. The greatest wear was observed in XAR 600 steel. Its wear in medium and heavy soil was similar and it was more than eight times greater than in light soil (Figure 10). The wear of the F-61 padding weld was different (Figure 10). Its intensity in light soil was twice as low than in the medium and heavy soils. The F-61 padding weld with an increased niobium content proved to be the least sensitive to a change in the soil mass grain size distribution (2.1×), followed by B27 post-martensitic steel with fine carbide grains within martensite (2.6×), nitride-bonded silicon carbide (6.5×) and steel with the structure of fine lath hardening martensite with evenly distributed areas of tempered martensite—XAR 600 (8.7×). The wear intensity for silicon carbide was the lowest in all soil types (0.0118 g/km). It was only slightly greater for the F-61 padding weld (0.0129 g/km). The steel wear intensity was more than six times greater than in silicon carbide: XAR 600—0.0698 g/km and B27—0.0727 g/km. 

**Figure 11:**
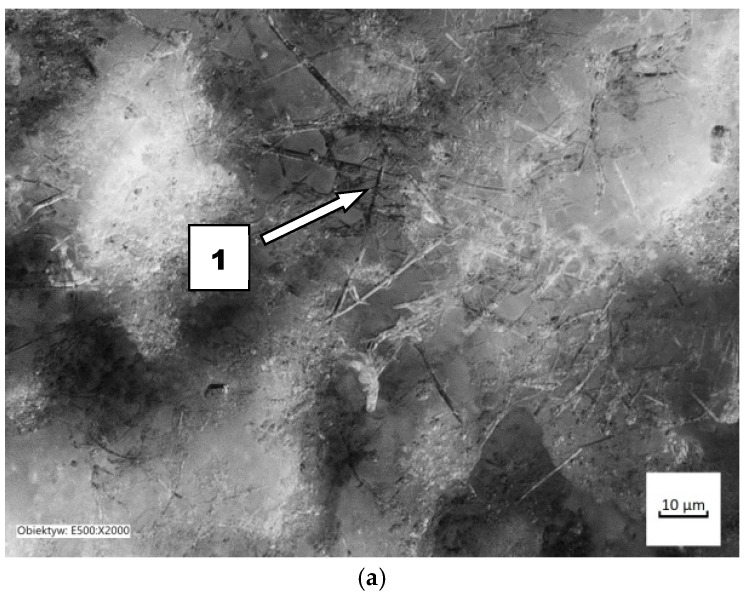

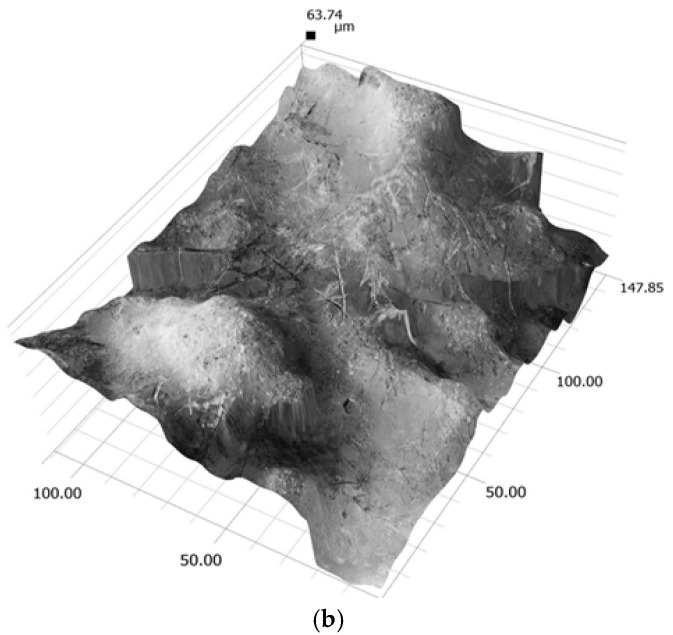
The friction surface condition after wear in light soil: (**a**) scratches and local surface chipping, magnitude 2000×; (1) micro-cutting; (**b**) friction surface profile.

The cause of these relationships should be sought in the way each material wears out. With regard to the presented methods of wear of ceramic materials, it should be stated that it has been confirmed that the basic method of wear is abrasive. When nitride-bonded silicon carbide wears out in light soil, loose grains of sand, moving freely around, usually scratch the friction surfaces. In the few cases when the possibility of movement was limited, micro-cutting took place, which resulted in chipping off the layer (Figure 11). It indicates that sand grains interact with the surface in a discrete manner. The dents in the worked surface were 67 µm deep, which was more than with any of the other soils under study.

**Figure 12:**
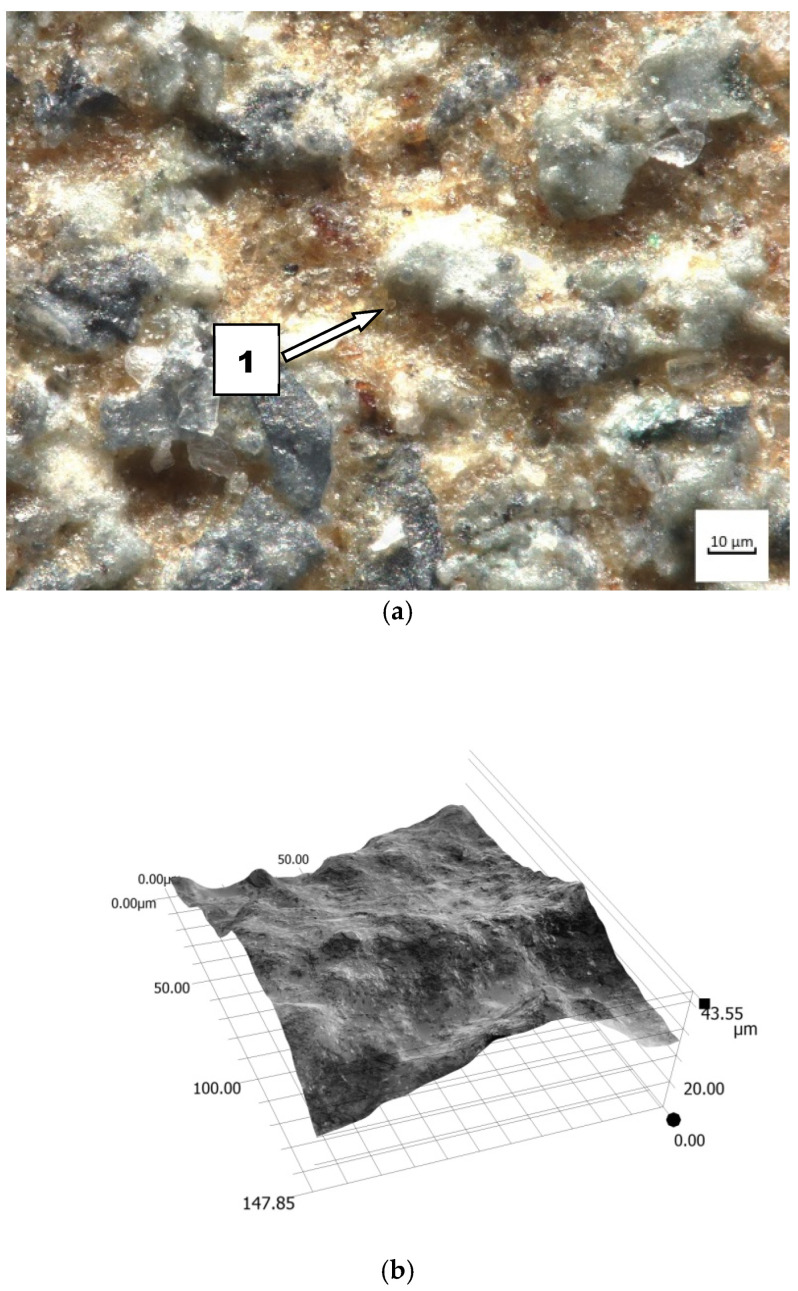
The friction surface condition after wear in medium soil: (**a**) view of coated grains of silicon carbide, magnitude 500×, (1) carbide grains before removal; (**b**) friction surface profile.

**Figure 13:**
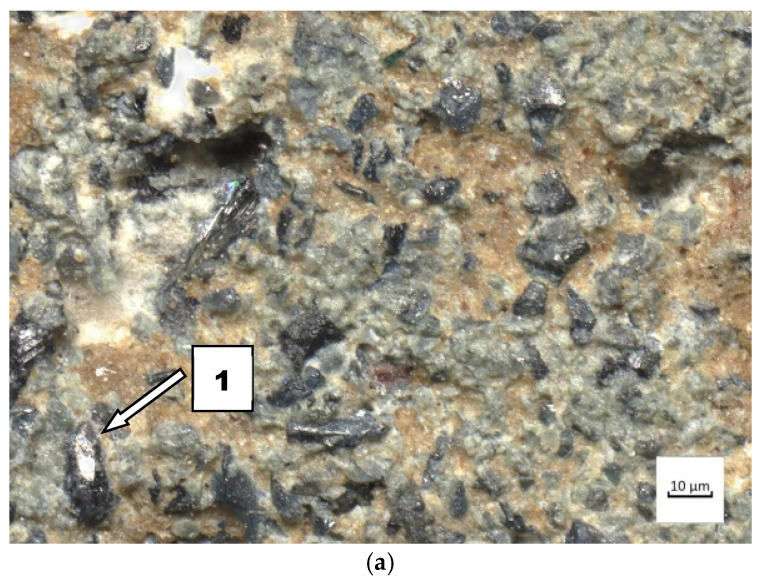

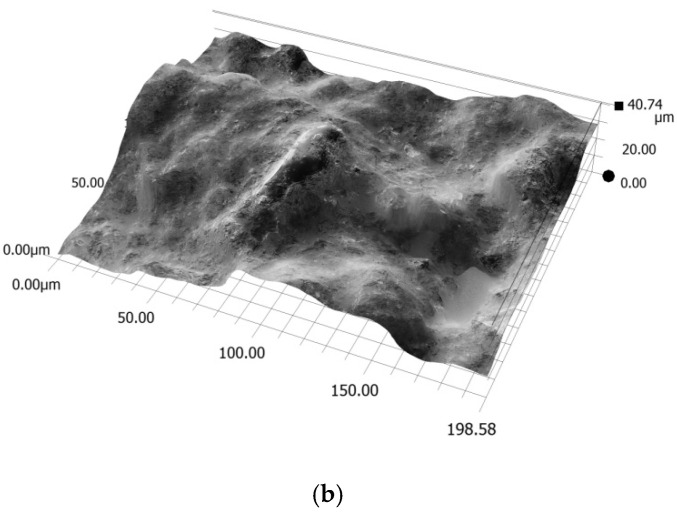
The friction surface condition after wear in heavy soil: (**a**) view of coated grains of silicon carbide, magnitude 150×, (1) the pores of the silicon carbide surface are grooved (wiped) over the entire surface; (**b**) friction surface profile.

A different course of wear can be observed in soil containing more dust and silt. The sand fraction comprises solely quartz SiO_2_. Dust and silt fractions contain mostly compounds of amorphous silica and silty minerals. The impact of silt and dust alone is negligible, but it intensifies when they are in combination with other fractions. When humid, these fractions act like an adhesive for quartz. The contact of soil mass with the material is discrete and the impact intensity depends on fixing grains in the soil mass. This implies a change in the nature of wear and, as a consequence, its intensification. The friction surface share increases (Figure 12). The porous surface of nitride-bonded silicon carbide (mean Ra—12.95 µm) is filled with an abrasive mixture, owing to which SiO_2_ grains have fewer degrees of freedom and the share of sliding friction increases at the expense of rolling friction. It is particularly manifest in the heavy soil impact (Figure 13). In this case, the soil contains much more silt and dust than sand (silt and dust—66.48%). The importance of hardness (2300 HV) is the greatest in the momentary contact of abrasive grains. This type of contact occurs in soil mass with loose grains of sand, where conventional wear takes place with simple destructive processes (micro-milling, ridging, scratching). The importance of the hardness of porous materials decreases in soils containing increased amounts of silty and dusty fractions. In heavy soil, silicon carbide is coated with fixed abrasive grains (sand) and less wear-resistant top layer components are removed. The surface in this soil was found to be less rough than in light soil. However, the number of dents increased, which confirms a different nature of wear, caused by a larger friction area. A larger area takes part in the wear process, which increases the wear intensity.

**Figure 14:**
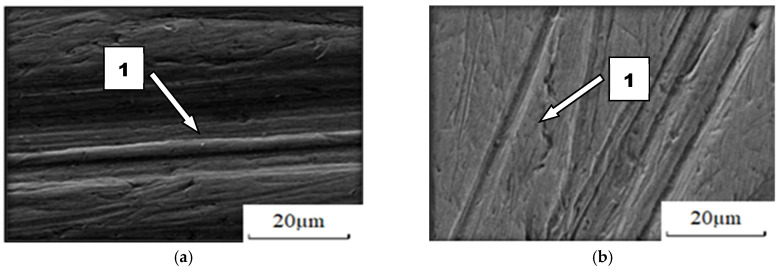

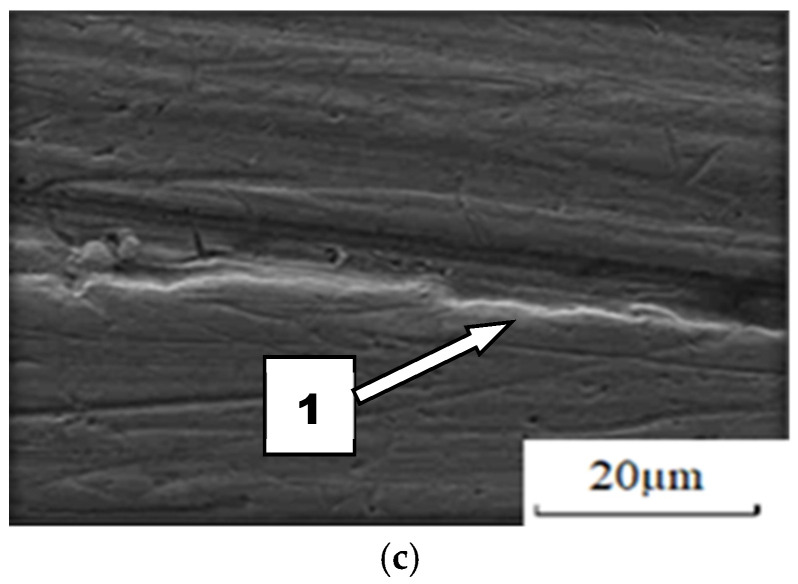
Condition of worn surface of XAR 600 steel in diverse soil conditions: (**a**) light soil, (1) furrowing; (**b**) medium soil, (1) furrowing; (**c**) heavy soil, (1) furrowing.

**Figure 15:**
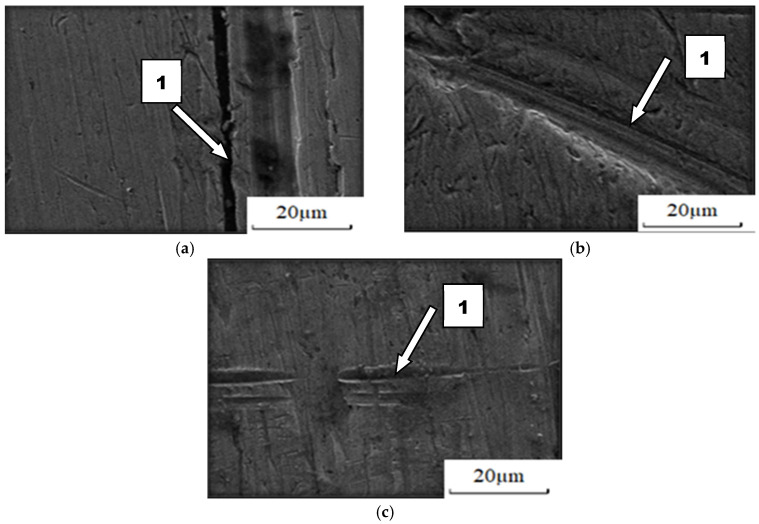
Condition of worn surface of B27 steel in diverse soil conditions: (**a**) light soil, (1) micro-cutting; (**b**) medium soil, (1) furrowing; (**c**) heavy soil, (1) furrowing.

**Figure 16:**
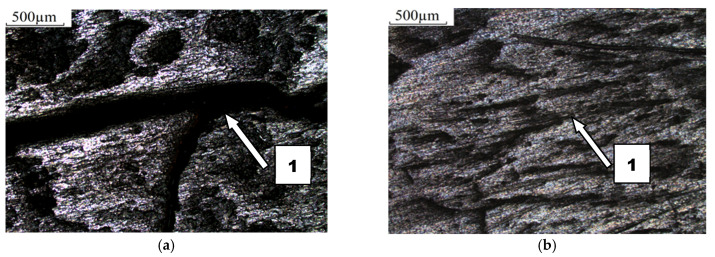

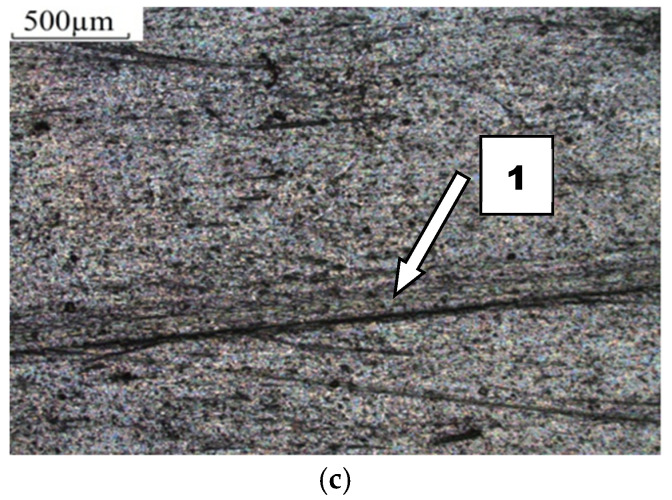
Condition of worn surface of F-61 padding weld in diverse soil conditions: (**a**) light soil, (1) gouging in discontinuities of the padding weld; (**b**) medium soil, (1) discontinuity of the padding weld; (**c**) heavy soil, (1) scratching.

The course of wear of boron-containing steels was similar in different soils (Figure 14 and Figure 15). Fatigue wear results from rounded SiO_2_ grains pressing repeatedly against the friction surface (dark areas). Multi-cycle wear consists of elastic strain, plastic strain, microvolume compression, structure deformation and unevenness shearing. Grooves are also visible, which are a consequence of the cutting impact of sharp edges of sand grains. This first phenomenon dominates in the friction process. As the share of dusty and silty fraction grows, the share of wear by grooving and micro-cutting also increases, which results in intensification of the wear process. The padding weld wear runs differently (Figure 16). Loose sand grains in light soil got into the surface discontinuities following padding, causing an increase in micro-cracks (surface penetration). This caused quick wear of the carbide matrix, with carbide particles being expelled from the top layer. As the share of fine fractions in soil grew, they penetrated surface discontinuities and protected it against an intensive impact of the sand fractions, resulting in scratches and local surface chip-offs on the surface worn in heavy soil.

The analysis of the top layer wear did not take into consideration the impact resistance of the tested materials. The steels and padding weld are capable of transferring lateral and dynamic loads [30]. For SiC, its common use is restricted by brittle cracking caused by mechanical loads. Cracking usually starts in material defects. Stable conditions of wear in natural conditions were ensured during the test. No chipping or cracking of the specimens with SiC was found. Therefore, it was confirmed that sintered nitride-bonded silicon carbide is highly resistant to abrasion in loose soils, but its resistance to brittle cracking is also increased.

The abrasion resistance index *K_b_* (Table 6) with respect to the wear of nitride-bonded silicon carbide shows that the wear resistance of the material was the highest in light soil containing loose grains of sand and was 1.2 times higher than the wear resistance of XAR 600 steel, 1.5 times higher than that of F-61 padding weld and more than eight times higher than that of B27 steel. Much higher wear resistance than SiC in the other soils was determined for the padding weld layer (from 1.8 times higher in medium soil to 9 times higher in heavy soil). Compared to steel, silicon carbide wore out less intensively in medium and heavy soil by a factor of nine (XAR 600 steel in medium soil) to 1.2 times (XAR 600 steel in heavy soil). Taking into account the mean wear, the wear of nitride-bonded silicon carbide was twice as small compared to special steels intended for working parts used in soil working. For padding weld containing C + Cr + Nb, its wear is three times less intensive than that of nitride-bonded silicon carbide.

The paper presents an analytical analysis of the wear of wear-resistant materials in abrasive soil based on the data obtained from empirical tests. In the case of such problems, new classes of solutions are available and commonly used on machine learning (ML) models, such as neural networks (NN) or regression trees [31]. The use of ML methods can overcome the limitations of empirical solutions when analytical solutions are not available and significantly change the way engineering problems are solved. The obtained results can form the basis for further research with the use of machine learning (ML) methods.

## 4. Conclusions and Discussion

Wear properties of nitride-bonded silicon carbide in abrasive soil mass depend on the soil grain size distribution. The best wear properties were determined in light soil. The wear resistance in this soil was 1.36 times higher than in medium soil and 6.5 times higher than in heavy soil. The course of wear of nitride-bonded silicon carbide and other construction materials in soil mass are described well by straight lines. Therefore, wear increase depends on the friction distance. These values are mostly decided by the manner of wear. When the soil contains large amounts of silt and dust, the friction area increases, which leads to softer particles of silicon carbide being chipped off. In light soil, local scratches and cracks occur, associated with the impact of loosely bound SiO_2_ particles. 

The best anti-wear properties, measured as the abrasive wear resistance index, were observed in the Fe-Cr-Nb padding weld applied onto 38GSA steel. Only in light soil was its wear greater than that of silicon carbide. The wear resistance index for steels resistant to abrasive wear was lower than that of nitride-bonded silicon carbide in every soil type.

The study findings showed good wear resistance of nitride-bonded silicon carbide in light and medium soil as compared to steel types commonly used to make soil working parts. For this application, silicon carbide can replace steel. However, one must stress that, for example, impact wear resistance of silicon carbide is much lower than that of the steel types under study. It limits the possibility of the widespread use of nitride-bonded silicon carbide to make soil working parts. 

## Figures and Tables

**Table 1:** Characteristics of soil abrasive mass.

Granulometric Groups	Fraction Diameter (mm)	Fraction Content (%)		
SAND	2.0–0.05	33.62	52.66	77.48
FINES	0.05–0.002	49.92	40.32	20.83
SILT	<0.002	16.56	7.02	1.69
Determined as per PN–EN ISO 14688–2(2006)		Ordinary soil—heavy soil	Light loam—medium soil	Loamy sand—light soil

**Table 2:** Chemical composition of the material under study.

Silicon Carbide SiC–A 80 (%)	
SiC + Si_3_N_4_	94
SiC + Si_3_N_4_ + Si_2_N_2_O	>95
Free Si	<0.5
Fe_2_O_3_	<0.2

**Table 3:** Properties of silicon carbide.

Maximum working temperature (°C)	1600
Open porosity (%)	18
Compression strength (MPa)	100
Resistance to thermal shocks (n/H_2_O)	>40
Abrasion resistance acc. to Böhme (cm^3^)	5.5
Roughness Ra (µm)	12.95
Apparent density (g/cm^3^)	2.5

**Table 4:** Characteristics of the chemical composition of the top layers of materials taken for tests.

XAR 600		B27		F-61	
C	0.37	C	0.27	C	5.2
Si	0.71	Si	0.25	Mn	0.4
Mn	0.98	Mn	1.2	Si	1.3
Cr	0.9	Cr	0.32	Cr	29
Mo	0.34	Mo	0.01	Nb	6.8
Ni	0.83	Ni	0.05		
B	0.003	B	0.002		
**Hardness**					
540 +/− 8 HBW		470 +/− 4 HBW		779 HV10	

**Table 5:** Mean mass wear of materials taken for the test.

Material	Mass Wear (g)					
	Light Soil	Standard Deviation	Medium Soil	Standard Deviation	Heavy Soil	Standard Deviation
SiC	0.0806	0.0337	0.1099	0.0386	0.5229	0.2004
XAR 600	0.3065	0.1090	1.9900	0.6761	1.9653	0.6780
B27	0.8957	0.3041	1.1395	0.3904	2.3278	0.7825
F-61	0.3889	0.1335	0.2004	0.0681	0.1889	0.0637

**Table 6:** Abrasive wear index *K_b_*.

	Light Soil		Medium Soil		Heavy Soil		Mean Value for the Three Soil Types	
	*K_b_*	Confidence Interval Limits	*K_b_*	Confidence Interval Limits	*K_b_*	Confidence Interval Limits	*K_b_*	Confidence Interval Limits
XAR600	0.8205	0.0338	0.1723	0.2095	0.8301	0.2101	0.5222	0.1511
B27	0.3059	0.2425	0.3279	0.1210	0.7637	0.0942	0.5559	0.1526
F61	0.6798	0.0413	1.7987	0.0211	9.0795	0.0197	3.0069	0.0273
SiC	1.0000	0.0083	1.0000	0.0112	1.0000	0.0515	1.0000	0.0237

## Data Availability

The data presented in this study are available on request. The presented data are not publicly available.
